# Immunization against an IL-6 peptide induces anti-IL-6 antibodies and modulates the Delayed-Type Hypersensitivity reaction in cynomolgus monkeys

**DOI:** 10.1038/srep19549

**Published:** 2016-01-19

**Authors:** Lucille Desallais, Caroline Bouchez, Hadley Mouhsine, Gabriel Moreau, Rojo Ratsimandresy, Matthieu Montes, Hervé Do, Françoise Quintin-Colonna, Jean-François Zagury

**Affiliations:** 1 Laboratoire Génomique, Bioinformatique et Applications, EA 4627, Chaire de Bioinformatique, Conservatoire National des Arts et Métiers, 292 rue Saint Martin, 75003 Paris, France; 2 Toxicologist, Nice, France; 3 Peptinov, 29 rue du Faubourg Saint-Jacques, Pépinière Paris Santé Cochin, Hôpital Cochin, 75014 Paris, France; 4 INSERM U970 PARCC (Paris Cardiovascular Research Center); Université Paris Descartes; Sorbonne Paris Cité; Paris, France

## Abstract

Interleukin-6 (IL-6) overproduction has been involved in the pathogenesis of several chronic inflammatory diseases and the administration of an anti-IL-6 receptor monoclonal antibody has been proven clinically efficient to treat them. However, the drawbacks of monoclonal antibodies have led our group to develop an innovative anti-IL-6 strategy using a peptide-based active immunization. This approach has previously shown its efficacy in a mouse model of systemic sclerosis. Here the safety, immunogenicity, and efficacy of this strategy was assessed in non human primates. No unscheduled death and clinical signs of toxicity was observed during the study. Furthermore, the cynomolgus monkeys immunized against the IL-6 peptide produced high levels of anti-IL-6 antibodies as well as neutralizing antibodies compared to control groups. They also showed an important decrease of the cumulative inflammatory score following a delayed-type hypersensitivity reaction induced by the Tetanus vaccine compared to control groups (minus 57,9%, *P* = 0.014). These findings are highly significant because the immunizing IL-6 peptide used in this study is identical in humans and in monkeys and this novel anti-IL-6 strategy could thus represent a promising alternative to monoclonal antibodies.

Interleukin-6 (IL-6) is a key messenger of immune responses and inflammation processes. It is produced by a wide variety of cell types such as lymphocytes, macrophages, neutrophils or fibroblasts. IL-6 is the major regulator of acute phase protein synthesis in response to injury^1^. It is also involved in the proliferation and differentiation of cytotoxic T cells by inducing the production of IL-2 and its receptor. The overproduction of IL-6 has been associated with many autoimmune diseases or cancers. IL-6 serum concentration is increased in sera of rheumatoid arthritis (RA)^2^, systemic sclerosis (SSc)^3^ and multiple myeloma patients^4^ and is correlated with disease severity^5^,^6^,^7^,^8^,^9^. Several studies have shown a protective effect of anti-IL-6 receptor (IL-6R) blockade strategies in mouse models of autoimmune diseases^10^,^11^. In humans, the pathological effects of IL-6 overproduction are specifically inhibited by tocilizumab, a commercialized monoclonal antibody directed against IL-6R, approved in 2009 for the treatment of rheumatoid arthritis, and in 2011 for the treatment of systemic juvenile idiopathic arthritis.

Anti-cytokine biologics (monoclonal antibodies and soluble receptors) have successfully been used to treat chronic inflammatory diseases since the late 90′s^12^. These biologics present however several drawbacks including primary and secondary resistances^13^,^14^, repeated injections and thus high compliance required from patients, side effects^15^, and prohibitive costs for patients and healthcare systems (up to $20,000 per patient per year). In order to overcome these disadvantages and to widen the therapeutic offer for the patients, new anti-cytokine targeted strategies are needed^16^. As an alternative and innovative strategy, our group has developed anti-cytokine peptide-based active immunization, where cytokine-derived peptides linked to a carrier protein are used as immunogens to induce anti-cytokine autoantibodies production. Our group recently demonstrated the efficacy of anti-IL-6 active immunization in the bleomycin mouse model of SSc^17^. In the present work, in order to adapt this strategy to human use, our aim was to test the hIS200-coupled peptide in cynomolgus monkeys as, in this species, this peptide sequence is identical to the human one. Therefore, we evaluated: 1) the safety of anti-IL-6 active immunization in cynomolgus monkeys; 2) the ability of the vaccine to induce a strong anti-cytokine antibody response; 3) active immunization efficacy in a delayed-type hypersensitivity (DTH) model^18^.

## Methods

### Molecular design, peptide synthesis and coupling

The hIS200 peptide was designed using the human IL-6/IL-6Rα/gp130 structure (PDB ID: 1P9M). This peptide was chosen in loops exposed to the protein surface, which correspond to a region involved in the interaction between the cytokine IL-6 and its receptor IL-6Rα.

The hIS200 peptide sequence is: _Acetyl+_ **C**_78_**ESSKEALAENNLNLPK**_94_CY.

The sequence in bold (**CESSKEALAENNLNLPK**) is strictly identical between human and cynomolgus monkey IL-6. Peptides were synthesized as previously described^17^ by Polypeptide Laboratories (Strasbourg, France). They were produced in a cyclized form by the formation of intramolecular disulphide bonds between cysteine residues and coupled with bis-diazobenzidine (BDB) to Keyhole Limpet Hemocyanin (KLH).

### Animals

Twelve cynomolgus monkeys (*Macaca fascicularis*) of adult age (between 4.5 and 5.5 years old) were separated into three groups: the first group received KLH only, the second group received a control peptide coupled to KLH and the third group was immunized with the hIS200 peptide coupled to KLH. Individual data of the animals are presented in Table 1.

Before inclusion in the experiment, each monkey received a complete health check including clinical examination, tuberculin testing, treatment against parasites and coprology tests. During the experiments, animals were housed in groups in dedicated pens at CIT (Centre International de Toxicologie) animal facilities (Évreux, France). The environment conditions were continuously recorded (temperature 22 ± 3 °C and relative humidity 50 ± 30%). The daily diet during the study consisted of commercial food pellets (Dietex France, SDS, Saint Gratien, France). In addition, a fruit supplement was given daily to each animal. Drinking water was provided *ad libitum*. The monkey W62010, from the hIS200-immunized group, received three sessions of non-steroidal anti-inflammatory drug (NSAID) and antibiotic drug administration (Finadyne^®^ and Suramox^®^ from days 9, 44 and 105) following a recurrent dental abscess (which started on day 9 and was not related to the immunization).

### Ethics

All study protocols and experimental procedures were approved by The CIT Ethical Committee and were carried out in accordance with the approved guidelines.

### Immunizations with the hIS200 peptide

**Figure 1:**
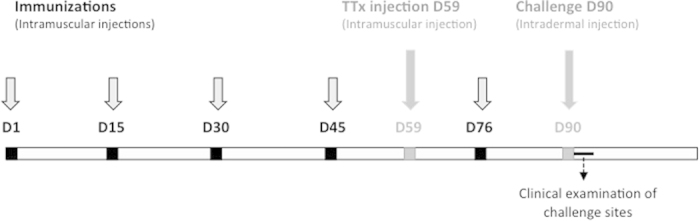
Study design. The monkeys received one priming (d1) and 4 boosters (d15, d30, d45, d76). The DTH reaction was evaluated on day 90, 30 days after the priming against TTx (day 59).

The study design is presented in Fig. 1.

Three groups of four monkeys were immunized five times intramuscularly into the right thigh, on days 1, 15, 30, 45 and 76. The first group, which received the control peptide (150 μg/monkey) conjugated to KLH (300 μg/monkey according to the manufacturer of the conjugate, Polypeptide Laboratories) and the second group, which received KLH only (Pierce, IL, USA), were used as negative controls. The third group of four monkeys was immunized with the hIS200 peptide (150 μg/monkey) conjugated to KLH (300 μg/monkey according to the manufacturer of the conjugate, Polypeptide Laboratories). Each treatment was emulsified in Montanide ISA 51 VG (Seppic, France). All monkeys received an injection of 0.3 ml on every immunization day.

### Clinical examinations

Each animal was checked for mortality or sign of morbidity at least twice a day during the study and clinical signs of systemic toxicity were checked. Body weights were also recorded once a week and rectal temperatures were measured before immunization, 6 h and 24 h after immunization on days 45 and 76.

### Dosage of anti-IL-6 and anti-Tetanus toxoid antibodies by ELISA

Blood samples were taken from the monkeys in pre-dose (day -8), before the first immunization and then every two weeks until the end of the study (total of 9 samples). The IgG response against human IL-6 and Tetanus toxoid (TTx) was measured by ELISA as previously described^17^. Briefly, 50 ng of human IL-6 (R&D Systems, Lille, France) or 50 ng of TTx (List Biological Laboratories, Campbell, USA) were adsorbed on microtitration plates (Nunc Maxisorp) overnight. After a saturation step, sera from immunized animals were serially diluted and added in coated wells. After wash, an incubation with polyclonal anti-monkey IgGs coupled to horseradish peroxidase (HRP) was performed. Plates were revealed with tetramethylbenzidine (TMB) and the reaction was stopped with 1 M sulfuric acid before reading on a spectrophotometer (Multiskan Ex, Thermo Scientific) at 450 nm. ELISA titers were expressed as those serum dilutions that lead to half-maximal OD_450_ (titer_50_).

### Purification of immunoglobulins from monkey sera

It was important to purify the serum immunoglobulins to avoid any non-specific serum interference in the IL-6 neutralization assay (see below). The total IgG fractions were purified from the sera of immunized monkeys using a protein A and protein G affinity chromatography (Biotem, Apprieu, France). In brief, 3 ml of each sera were diluted five times in PBS 1X sodium EDTA 5 mM and injected on the column. After washes, elution of bound IgGs was accomplished with ammonium acetate 500 mM pH3 and neutralized with Tris 1 M pH 8,8. IgG fractions were then concentrated by centrifugation followed by two dialysis of two hours in PBS at 4 °C. Final volume of purified IgGs was 3 ml.

### IL-6 neutralization assay

This neutralization assay is based on the inhibition of the binding of IL-6 to its receptors, in an ELISA-derived assay. Microtitration plates were coated with 300 ng/ml of recombinant human gp130 Fc chimera protein (R&D Systems, Lille, France) in PBS overnight at 4 °C. hIL-6 at 1,3 ng/ml and hIL-6Rα at 200 ng/ml (R&D Systems, Lille, France) were preincubated with the antibodies purified from the sera of immunized animals (at a dilution of 1:3) for two hours at 37 °C. After a saturation step with PBS containing 2% bovine serum albumin, 100 μl of the mix were added to the coated plates and incubated overnight at 4 °C. The plates were then incubated with 200 ng/ml of hIL-6 biotinylated antibody (R&D Systems, Lille, France) for two hours at 37 °C. After wash, plates were incubated with avidin-HRP (1:500) for 30 minutes at 37 °C and were revealed with 100 μl of TMB solution. The reaction was stopped with 50 μl of 1M sulfuric acid solution. Absorbance was measured at 450 nm.

### Induction and evaluation of the DTH response in monkeys

The Tetanus vaccine (Sanofi Pasteur, France) was administered by intramuscular injection into the left thigh of the monkey on day 59, at a dose-volume of 0.5 mL per animal.

A challenge was performed by intradermal injection on day 90 on two sites of the back of each animal with 0.05 mL of Tetanus vaccine. Animals were slightly anesthetized before the injections. Skin inflammatory reactions at the challenge sites were recorded once daily for each animal before administration, 24 h, 48 h and 72 h after challenge. Three parameters were evaluated at each challenge site: erythema, dermal thickening and nodules. A score based on the incidence of the three-recorded parameters was used, with a 0 if the parameter was absent of the two challenged sites and a 1 if the parameter was present in at least one of the challenged sites. At each time point, the score for each animal was thus comprised between 0 and 3. The final score given to each animal corresponded to the sum of the scores obtained at 24 h, 48 h, or 72 h. The score for each group was the sum of the scores of the group animals (max = 36). Additionally, the size of the nodules was also measured using a caliper on day 93.

### Serum levels of IL-6

Serum levels of IL-6 were measured in all monkeys, by using U-CyTech sandwich old world monkey IL-6 ELISA kits with a detection limit of 5 pg/ml (U-CyTech Biosciences, Utrecht, The Netherlands).

### Statistics

A Fisher’s exact test was used to compare the scores of the inflammatory response in the various groups of monkey. Mann-Whitney tests were used to compare IL-6 levels between the different groups. *P* values ≤ 0.05 were considered significant.

## Results

### No adverse event observed during the monkey study

**Figure 2:**
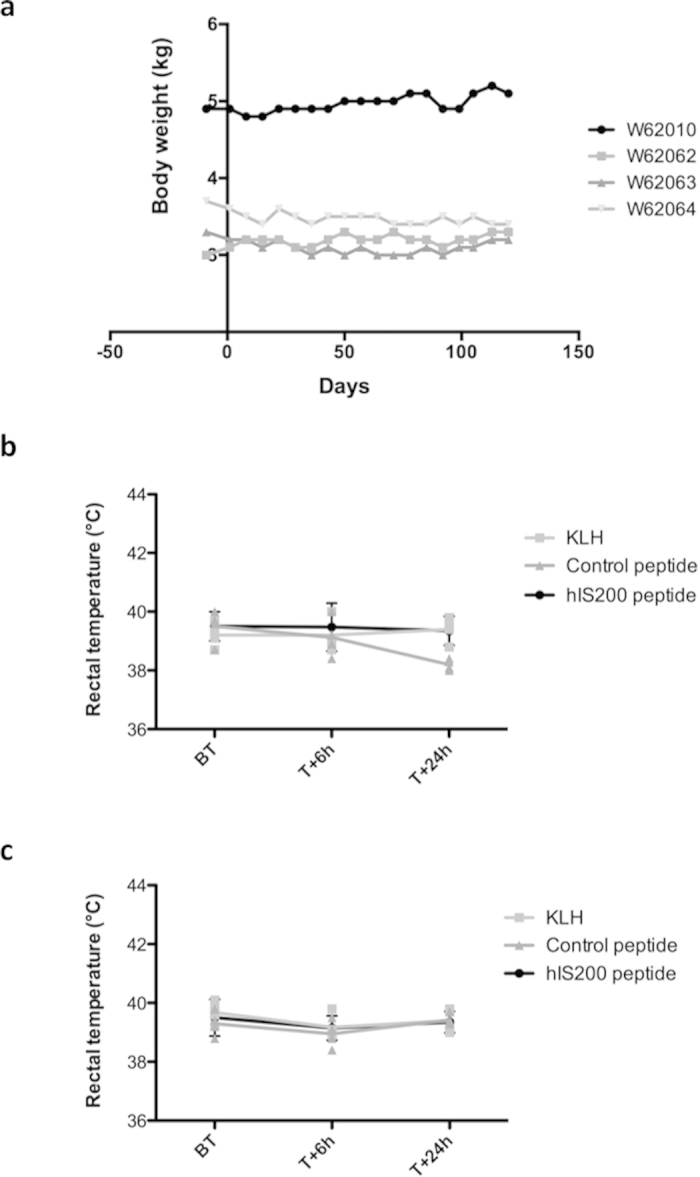
(**a**) Evolution of body weight (kg) in the hIS200-immunized group of monkeys. (**b**) Rectal temperature at week 7. (**c**) Rectal temperature at week 11. BT: before treatment, T + 6 h: 6 hours after treatment, T + 24 h: 24 hours after treatment.

During the whole study (4 months), monkeys were monitored for potential side effect reactions. No unscheduled death and no clinical signs of systemic toxicity were recorded during the study. Some minor local reactions (nodules) were observed at the injection sites in some animals from all groups. They were considered to be part of the immunologic reaction following the immunization. No significant weight loss was observed in the hIS200-immunized group during the study (Fig. 2a) nor in the control groups (data not shown). Moreover, no significant increase in the rectal temperature was recorded in any group, following injections with the peptide immunogens in week 7 (fourth injection) and week 11 (last injection) (Fig. 2b,c).

### Production of anti-IL-6 antibodies in monkeys

**Figure 3:**
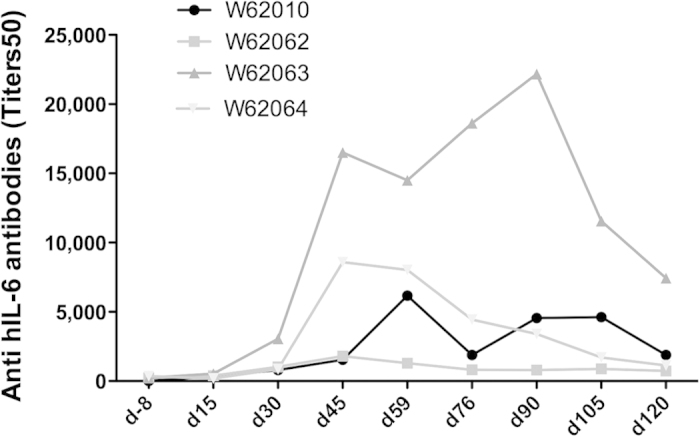
Kinetics of the anti-IL-6 antibody production in the cynomolgus monkeys immunized against the hIS200 peptide immunogen. The control groups did not exhibit any antibodies against IL-6 detected by ELISA and are thus not shown.

A high production of antibodies recognizing human IL-6 was measured in all four hIS200-immunized monkeys (Fig. 3), showing that the self-tolerance was overcome. However, antibodies titers decreased at the end of the study without further boosts. No anti-human IL-6 (hIL-6) antibodies were found in the control groups (data not shown).

### Presence of anti-IL-6 neutralizing antibodies

**Figure 4:**
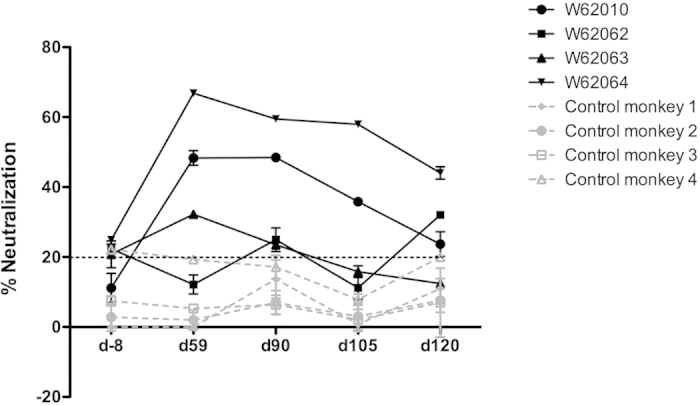
Neutralizing capacity of the antibodies purified from sera of the immunized monkeys at d-8, d59, d90, d105 and d120. The four hIS200-immunized monkeys (in black) exhibit a significant IL-6 neutralization capacity higher than the background, while the neutralizing capacity of the control monkeys (in grey) is always below background value (threshold of 20% marked by the dotted line). Only one control group (KLH) is shown for clarity of the figure (the values of the other control group are also all below background value).

The IL-6 neutralization capacity of the antibodies induced by the immunization of monkeys was assessed by measuring the inhibition of binding of IL-6 to its receptors IL-6Rα/gp130. In order to avoid any non-specific interference of serum, the IgG antibodies of the immunized monkeys were purified. Neutralizing antibody responses were observed in all hIS200-immunized monkeys with a maximum neutralizing capacity on d59 which decreased over time. No neutralization was observed for the control groups (Fig. 4). The neutralizing capacity in the hIS200-immunized monkeys along time followed a similar evolution as the anti-IL-6 antibody titers_50_ (see Supplementary Fig. S1 online).

### Significant decrease of the DTH response clinical signs following immunization against the hIS200-coupled peptide

**Figure 5:**
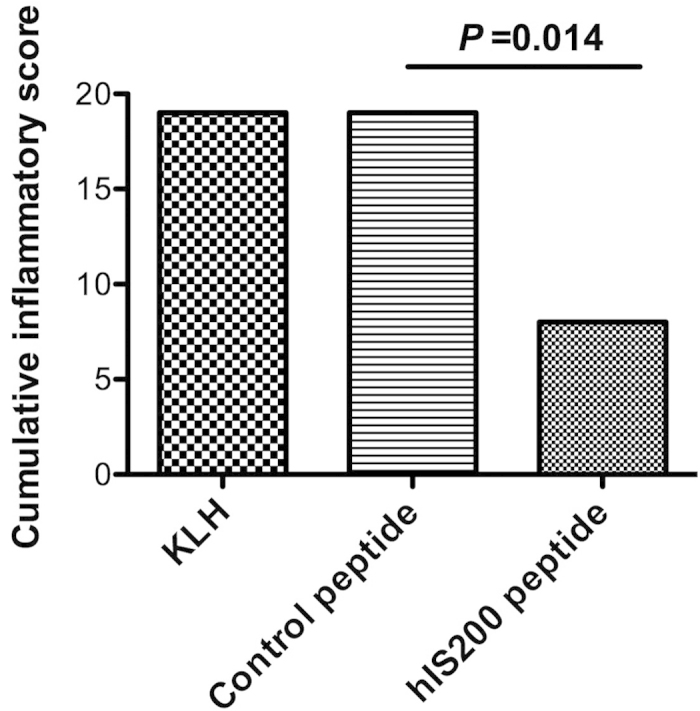
Cumulative inflammatory score of the local reactions of the immunized monkeys, based on erythema, dermal thickening, and nodules at the sites challenged with 0.05 ml of TTx vaccine.

Following the Tetanus toxoid challenge performed on day 90, all animals developed a DTH skin reaction at injection sites. In spite of the small size of the groups, the cumulative inflammatory score (erythema, dermal thickening, and nodules) for the hIS200-immunized group was significantly lower (*P* = 0.014) compared to control groups immunized against a control peptide or against KLH (Fig. 5). In control groups, erythema grade and duration were increased compared to hIS200-immunized group as well as dermal thickening duration. Furthermore, the nodules observed in the hIS200-immunized group were too small to be measured compared to controls (Table 2). Individual DTH inflammatory score of the local reactions for each monkey is presented in Supplementary Table S2. No correlation was observed between anti-IL-6 titers and the observed reduction in the DTH response among the four hIS200-immunized monkeys.

The TTx antibody response was also evaluated and was very similar in the three groups (see Supplementary Fig. S3 online). The reduced clinical scores observed in the test group is thus likely not due to a decreased priming against TTx.

### Mean serum IL-6 levels increase after active immunization

**Figure 6:**
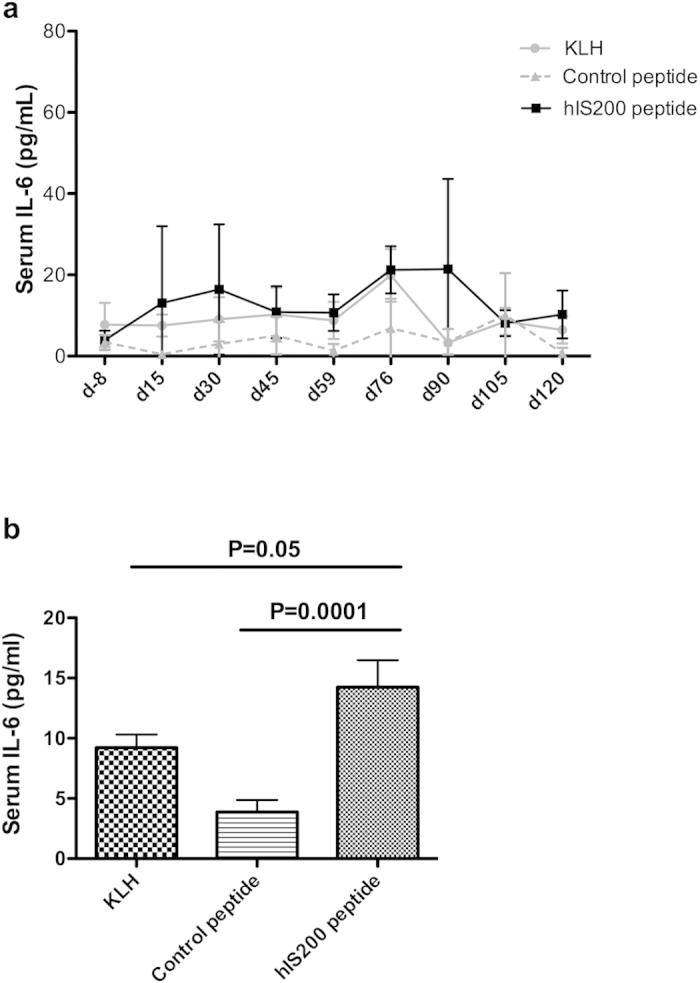
(**a**) IL-6 levels in the serum of monkeys were measured by ELISA. Data are presented as mean ± SD for each group at each time point. The mean level is always slightly higher in the hIS200-immunized group at each timepoint. (**b**) Analysis by the Mann-Whitney test shows that the mean IL-6 concentration over time is significantly higher in hIS200-immunized monkeys compared to the two control groups.

The mean serum IL-6 concentration at each time point (e.g. test samples vs. control samples at day 15, or at day 30, etc.) showed no significant difference between the test group and the control groups. However, one can observe that the mean serum IL-6 concentration for all time points was higher from day 15 to day 120 in the test group than in the two other groups (Fig. 6a). Using a global statistical approach over time (Mann-Whitney test, see methods), we have found that the mean IL-6 concentration was in fact significantly higher in the hIS200-immunized group compared to the control peptide group (P = 0.0001) and compared to the KLH control group (P = 0.05) (Fig. 6b).

## Discussion

This study is the first one showing the clinical efficacy of an anti-IL-6 immunization in non-human primates. Some other groups have used a similar approach with analogues of IL-6 and showed efficacy in murine models of multiple myeloma^19^, collagen induced arthritis (CIA)^20^, and in hIL-6 transgenic mice^21^, but this “IL-6 analogue” approach was not tested in monkeys. Our group has previously shown that immunization against a murine peptide of IL-6 induced the production of anti-IL-6 antibodies was able to reduce bleomycin-induced fibrosis in mice^17^. In the present study, in order to translate this strategy into humans, we evaluated the safety, immunogenicity and efficacy of anti-IL-6 active immunization in cynomolgus monkeys. Cynomolgus monkeys were of particular interest because the sequence of the hIS200 peptide (derived from IL-6) is 100% identical between human and cynomolgus monkey IL-6. We observed that the IL-6 peptide was well tolerated, highly immunogenic in monkeys, and capable of eliciting anti-IL-6 neutralizing antibodies. This antibody production was reversible and regular booster vaccines are required to sustain the antibody levels, which constitutes a safety feature. Most importantly we showed that anti-IL-6 active immunization had *in vivo* effects since it was able to modulate the inflammatory reactions following a DTH response induced by TTx. Indeed, the results showed a significant 57.9% decrease of the inflammatory reactions in the IL-6-immunized group compared to controls. As described by Galle *et al.*^20^ with IL-6 analogues immunization in a murine model of CIA, we did not observe a direct correlation between anti-IL-6 titers and the reduction of the clinical score following TTx challenge within the IL-6 immunized group. Furthermore, we have shown that the response to TTx was very similar in the hIS200-immunized group and in the control groups suggesting that the decrease of DTH clinical scores in hIS200 immunized monkeys was not induced by a decreased priming against TTx, but rather by the effective neutralization of IL-6 at day 90 due to the immunizations.

We also observed that the mean serum IL-6 concentration at each time point showed indeed no significant difference between the test group and the control groups nor did the levels of C-reactive protein (CRP) (data not shown). However, the mean IL-6 serum concentration for all time points was always higher from day 15 to day 120 in the test group than in the two other groups. Using a global statistical approach over time, we have found that the mean IL-6 concentration was in fact significantly higher in the hIS200-immunized group compared to the control peptide group (P = 0.0001) and compared to the KLH control group (P = 0.05). Such an increase of IL-6 serum concentration was observed with the anti-IL-6 monoclonal antibody Sirukumab and was explained by immune complexes^22^. The increase of IL-6 concentration was also observed in patients treated with Tocilizumab^23^, suggesting that this increase is well tolerated in patients treated by anti-IL-6 biologics. The CRP concentration was low in the three monkey groups of the study since they were healthy monkeys with no disease. Since the CRP values were low in all groups to begin with, it could not be reduced more by the vaccine in the hIS200-immunized group. A decrease of CRP levels has been observed in patients treated by anti-IL-6 biologics^24^,^25^ because they initially had a chronic inflammatory disease with high levels of CRP.

Our results confirm that active anti-cytokine immunization may be an efficient alternative to anti-cytokine monoclonal antibodies. Compared to the infusion of monoclonal antibodies, the advantages of the anti-cytokine vaccines are manifolds: no anti-idiotypic response meaning less secondary resistance, decrease of the costs considering the small quantities of required immunogen, simplicity of production, and simplicity of administration to the patients. A few examples of vaccines based on whole-cytokine immunogens targeting pro-inflammatory cytokines have been described in mice with IL-1^26^, IL-6^20^,^21^, IL-17^27^,TNFα^28^,^29^,^30^, and also in human clinical trials conducted against the IFNα^31^ and TNFα^32^ cytokines. However, there may be several advantages for the use of peptide immunogens over whole-cytokine immunogens: simplicity of production, better characterization of the immunogenic epitopes with less risk of T-cell immunity, cross-reactivity with other proteins or facilitating antibodies. A few examples of successful active immunization against cytokine-derived peptides have been described in mice models of inflammatory diseases with peptide immunogens from TNFα^33^, IL-1β^34^, IL-23p19^35^, and IL-6^17^.

In conclusion, this work strengthens the previous results obtained with a peptide immunogen from IL-6 in mice^17^ and shows for the first time, the feasibility of this original peptide-based strategy in non-human primates, paving the way for its future use in humans.

## Additional Information

**How to cite this article**: Desallais, L. *et al.* Immunization against an IL-6 peptide induces anti-IL-6 antibodies and modulates the Delayed-Type Hypersensitivity reaction in cynomolgus monkeys. *Sci. Rep.* **6**, 19549; doi: 10.1038/srep19549 (2016).

## Supplementary Material

Supplementary Information

## Figures and Tables

**Table 1:** Individual data of the monkeys.

Animal	Gender	Age (years)	Starting weight (kg)	Treatment
W62003	Male	5.2	3.97	Control peptide
W62004	Male	5.5	4.15	
W62053	Female	5.1	3.39	
W62054	Female	5.3	2.81	
W62009	Male	5.3	5.9	KLH
W62059	Female	5.1	2.47	
W62060	Female	5	3.12	
W62061	Female	4.9	2.74	
W62010	Male	5.4	4.6	hIS200 peptide
W62062	Female	5	3.16	
W62063	Female	5.1	3.34	
W62064	Female	5	4.12	

**Table 2:** Local skin reactions at challenge sites injected with 0.05 ml of Tetanus vaccine (mean for 2 sites: total of 8 sites for 4 animals/group).

Group	Control peptide	KLH	hIS200 peptide
*Erythema* (a) Grades(b) Duration	1–2 1.8	1–2 2	11
*Dermal thickening* (a) Grades(b) Duration	12	11.7	11
*Nodules* (c) Mean size	4.7	5.6	nm

(a): Grade of erythema and dermal thickening during the DTH evaluation (day 90 up to day 93), 1: very slight; 2: well-defined. (b): Mean duration (days) regarding DTH evaluation. (c): Mean size of nodules (mm), nm: non measurable.
