# Supplementary material for: Immunization against an IL-6 peptide induces anti-IL-6 antibodies and modulates the Delayed-Type Hypersensitivity reaction in cynomolgus monkeys
Source: Sci Rep. 2016 Jan 19;6:19549. doi: 10.1038/srep19549 (PMC4726013; doi:10.1038/srep19549)
Supplement: Supplementary Information [file srep19549-s1.doc]

Immunization against an IL-6 peptide induces anti-IL-6 antibodies and modulates the Delayed-Type Hypersensitivity reaction in cynomolgus monkeys

Lucille Desallais^1^, Caroline Bouchez^2^, Hadley Mouhsine^1^, Gabriel Moreau^3^, Rojo Ratsimandresy^3^, Matthieu Montes^1^, Hervé Do^3^, Françoise Quintin-Colonna^4^, Jean-François Zagury^1*^.

^1^Laboratoire Génomique, Bioinformatique et Applications, EA 4627, Chaire de Bioinformatique, Conservatoire National des Arts et Métiers, 292 rue Saint Martin, 75003 Paris, France.

^2^Toxicologist, Nice, France.

^3^Peptinov, 15 rue Ambroise Thomas, 75009 Paris, France.

^4^INSERM U970 PARCC (Paris Cardiovascular Research Center); Université Paris Descartes; Sorbonne Paris Cité; Paris, France.

**Email addresses:** [lucille.desallais@gmail.com](mailto:lucille.desallais@gmail.com), [caro.bouchez@gmail.com](mailto:caro.bouchez@gmail.com), [hadleymouhsine@gmail.com](mailto:hadleymouhsine@gmail.com), gabriel.moreau@peptinov.fr, [rratsima@gmail.com](mailto:rratsima@gmail.com), [matthieu.montes@cnam](mailto:matthieu.montes@cnam).fr, [herve.do@peptinov.fr](mailto:herve.do@peptinov.fr), [fquintin-colonna@vet-alfort.fr](mailto:fquintin-colonna@vet-alfort.fr)**,** jean-francois.[zagury@cnam.fr](mailto:zagury@cnam.fr)

***Corresponding Author**: Prof. Jean-François Zagury, MD, PhD

**Telephone**: +33 1 58 80 88 20

**E-mail**: jean-francois.zagury@cnam.fr

**
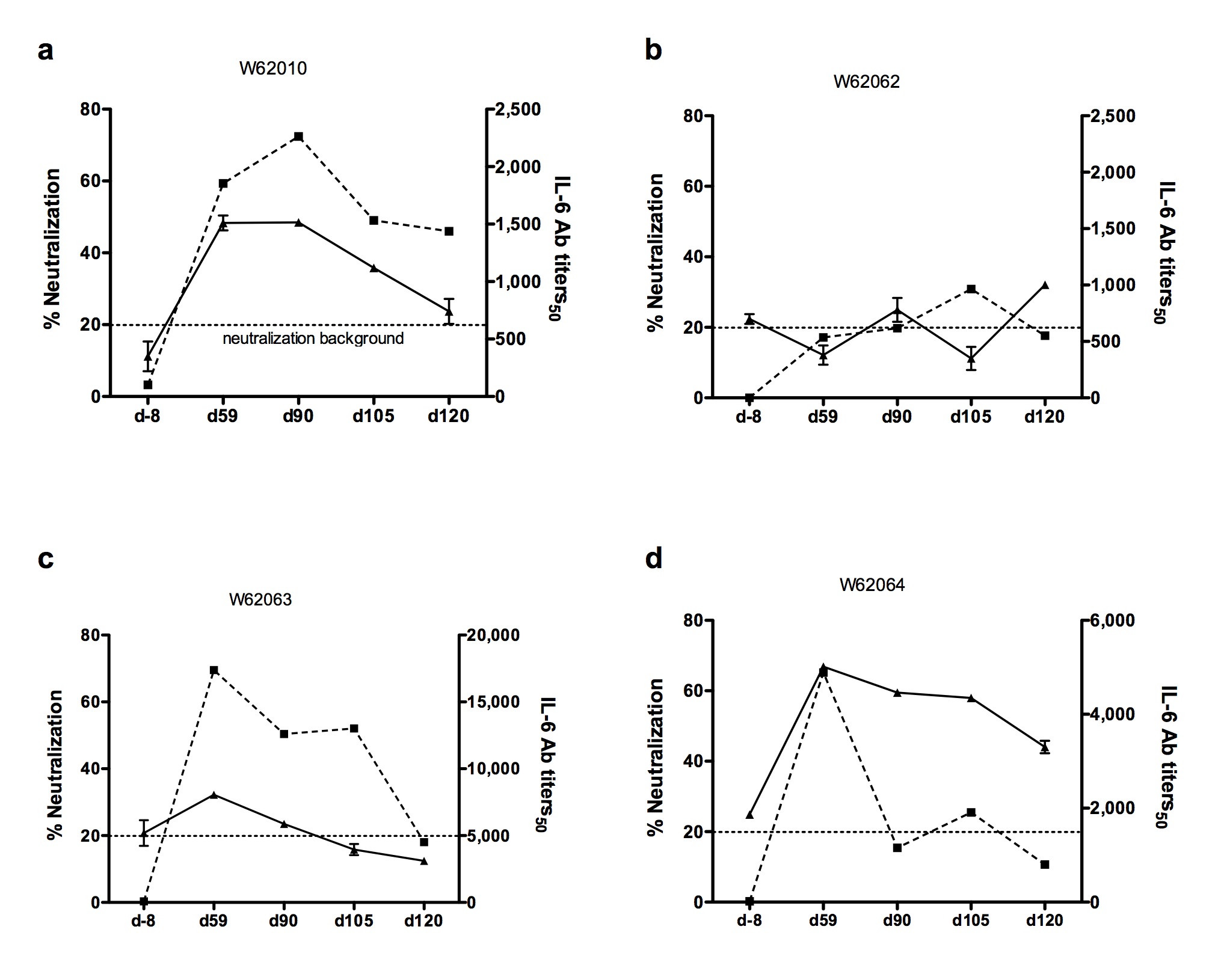
**

**Supplementary Figure S1:** Similar evolution of anti-IL-6 antibody titers_50_ (solid lines) and anti-IL-6 antibody neutralizing capacity (dashed lines) in the four hIS200-immunized monkeys.

| **Treatment** | **Animal** | **Inflammatory score** |
| --- | --- | --- |
| Control peptide | W62003 | 6 |
|  | W62004 | 3 |
|  | W62053 | 6 |
|  | W62054 | 4 |
| KLH | W62009 | 5 |
|  | W62059 | 3 |
|  | W62060 | 8 |
|  | W62061 | 3 |
| hIS200 peptide | W62010 | 4 |
|  | W62062 | 1 |
|  | W62063 | 1 |
|  | W62064 | 2 |

**Supplementary Table S2:** Individual inflammatory score of the local reactions for each monkey following TTx challenge at day 90.

**
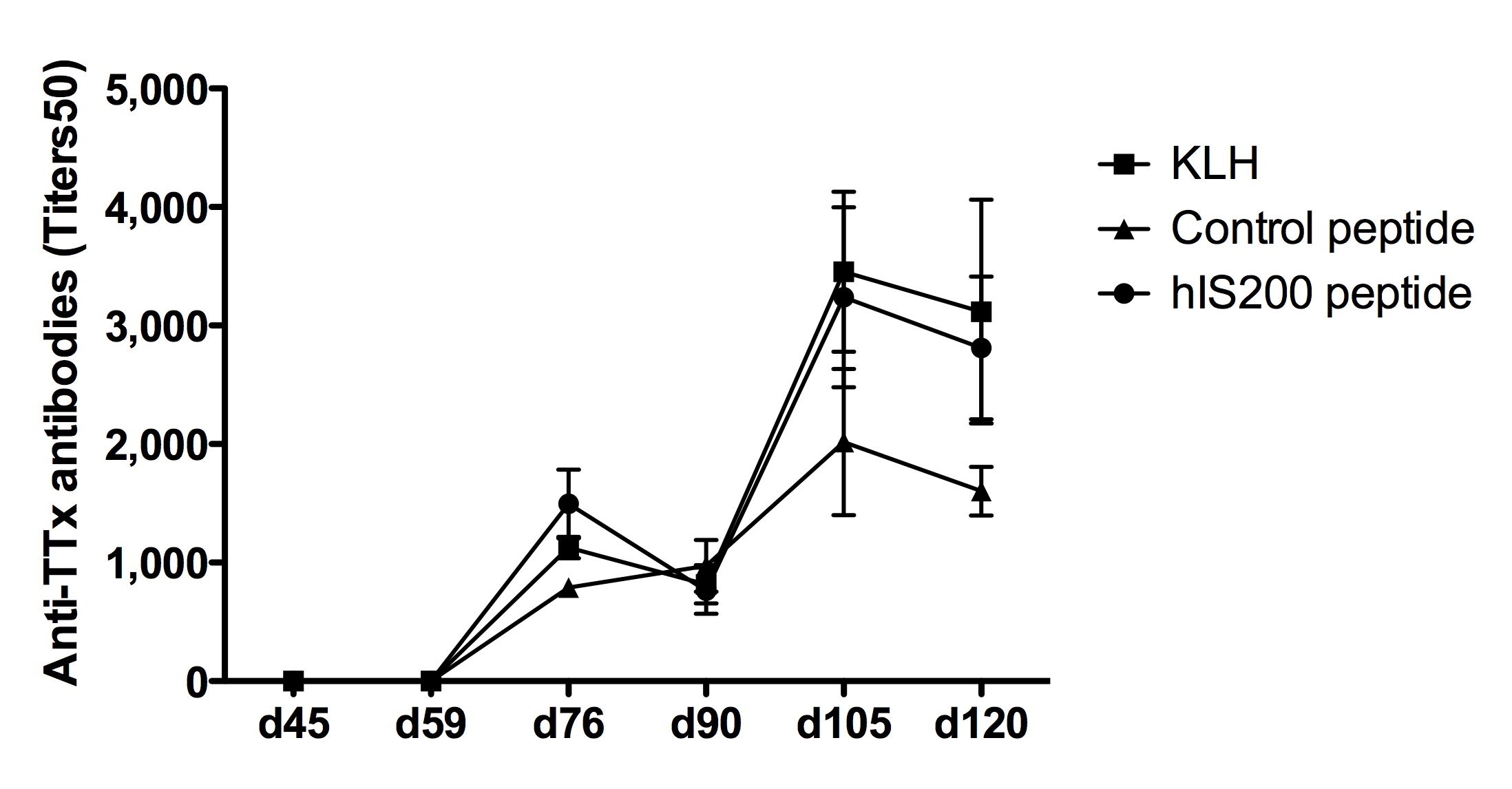
**

**Supplementary Figure S3:** Kinetics of the anti-TTx antibody production in all groups of immunized monkeys. The kinetics is similar in the three immunized groups.
